# COVID-19 pandemic and suicides in Nepal: Way forward for prevention

**DOI:** 10.3126/nje.v11i4.41116

**Published:** 2021-12-31

**Authors:** Indrajit Banerjee, Jared Robinson, Brijesh Sathian, Indraneel Banerjee

**Affiliations:** 1, 2 Sir Seewoosagur Ramgoolam Medical College, Belle Rive, Mauritius; 3 Geriatric and long term care Department, Rumailah Hospital, Hamad Medical Corporation, Doha, Qatar; 4 Consultant Uro oncologist and Robotic Surgeon, Apollo multi speciality Hospitals, Kolkata, West Bengal, India

## Background

The SARS-CoV-2 pandemic has firmly rooted itself within our countries, communities, homes and now everyday lives. The impact of this global pandemic is immeasurable as it is catastrophic in nature and involves both a human and financial loss. The effect thereof is boundless and reaches far beyond the mere closure of international boarders, nationwide lockdowns and supply chain interruptions. This worldwide pandemic has come at a real human cost, with millions now paying the ultimate price of losing their lives. The deadly and destructive nature of this virus extends far beyond the conventional physiological illness and subsequent morbidity induced on the human body, but has also exerted tremendous amounts of psychological stress which has now been seen to have driven some individuals to take their own lives. The impact and degree to which the most vulnerable members of our society are exposed to such thoughts and actions must be attained and better understood so as to better safeguard the mental health of all, but particularly those who are at their most fragile [1-3].

### Mental health and COVID-19

The novelty of the SARS-CoV-2 virus and lack of a specific and effective treatment, coupled with its intense predilection to infect and kill individuals with concomitant comorbidities led to large scale national and international lockdowns in order to best curtail the spread of the virus. These lockdowns further composed of self-isolation and isolation of individuals mandated by government. The inherent issue with this method of breaking the disease transmission chain is that often those whom are most vulnerable in both a physical and mental sense are most gravely affected by such policies. Besides the physical aspect of separation and isolation, other stressors and triggers of anxiety are elevated and precipitated by events such as lack of access to food, medical care and drugs [4,5].

The subconscious pressure and stress induced by such a global event, super-added to the newly found anxiety and super-charged isolation can make for a melting pot of emotions and mental instability which does not only affect individuals with good mental health, but can have a disastrous effect on those with prior mental health diseases and afflictions. The effects of such lockdowns and the COVID-19 pandemic as a whole on the mental health of individuals has now become prevalent as a rising number of suicides have been recorded, the fear is that this may only be an iceberg effect with many more individuals on the verge [6,7].

Various countries and nations have been adversely affected by COVID-19 in various manners spanning from the realm of economics, tourism, development, social wellbeing, enterprise to mental health with increasing warning signs that another epidemic (poor psychosocial and mental health leading to suicides and self-harm) may be stemming from the roots of this global viral pandemic. The multifactorial manner in which COVID-19 engulfs our lives via massive the lockdowns and the fear of death induces such a damaging cycle to the mental health of all. One such nation who seems to be particular hard hit by both COVID-19 and the mental health implications thereof is the mountainous country of Nepal [8].

Every year, around 700,000 people die by suicide globally. Low- and middle-income nations account for 77 percent of global suicides [9]. According to the World Health Organization, In 2016, WHO predicted Nepal's age-standardized suicide rate as 9.6 per 100,000 [10]. In a systematic review and meta-analysis, Farooq et al found that the pooled prevalence of suicidal ideation during COVID-19 was 12.1%. (CI 9.3-15.2). Low social support, increased physical and mental exhaustion, and lower self-reported physical health among frontline medical staff, insomnia, isolation and fatigue, loneliness, and psychological issues were the main risk factors for suicide ideation [11].

### Nepalese Mental health during COVID-19

The first case of COVID-19 in Nepal was recorded on the 13th of January 2020, with cases rising rapidly subsequently to this index case [12]. In light of this pandemic the Nepalese government enforced a nationwide full lockdown to try curtail and best curb the spread of the deadly virus. The Nepalese lockdown commenced from the 24th of March 2020 to September 2020. The lockdown had a plethora of effects on the nation, however some of the darker truths and effects thereof are being exposed now [12].

A cross sectional study conducted by Shrestha R, et al. on the impact of COVID-19 on both self-harm and suicide via emergency room visits to Dhulikhel hospital-Kathmandu University Hospital (DH-KUH) during the COVID-19 pandemic described that a total of 125 suicide and or self-harm cases were reported to the (ED) Emergency Department from March 24 to June 23, 2020. This was a raise in suicide and self-harm by 44% and 71.9% when compared over the lockdown period as when compared to self-harm and suicide statistics collected in 2019. It was discovered that organophosphate poisoning was the most common and preferred method used by patients, with the female gender being the most predominant gender in all three periods with a mean age of 32 years. The importance of this study is multifaceted as it both shows the negative effect of COVID-19 on mental and simultaneously exposes the hidden dimension of looming poor mental health circulating within the country. This subsurface level of poor mental health has been exposed through the precipitating factor of COVID-19 [8].

Further research into the suicidality and current impact of COVID-19 on the mental health of those in Nepal conducted by Acharya SR, et al. has highlighted the significant and dire situation that Nepal is facing as an average of 18 suicides a day occurred throughout the lockdown period. Numerous cases have also been reported where villagers committed suicide via hanging in order to protect their fellow kinsman from the virus as they feared they had contracted COVID-19. It is therefore clear that fear, depression, anxiety and a lack of knowledge coupled with lockdown and an obvious pre-existing mental fragility within the population precipitated the massive rise in suicide and self-harm [13].

### Prevention

Suicide and self-harm (SH) are both a serious public health and social issue. It is however preventable via the use of timely, evidence-based and many times low-cost interventions and therapies. The current situation depicted Nepal shows a true indicator of the mental health of the nation, as a precipitating factor (i.e., the extreme stress of COVID-19 and the lockdown) has exposed the submerged “ice-berg” phenomenon of disease. In the case of the developing nation of Nepal it is vital for further funding and development from various government stakeholders to coalesce in order to better curb such events and improve the overall mental health of the Nation. This will need a multifaceted and inter-related public health program initiated across the board from school education and counselling to the elderly. A vital factor for preventing such suicides is further education of the public on the virus as well de-escalation of mass pandemonium and panic. In conjunction with education, a vast amount of energy and time must be spent on breaking the social stigmas surrounding mental health, particularly in such a developing nation as Nepal where such mental issued are not often social understood and or accepted [14, 15].

## Conclusion

It is evident that COVID-19 and the lockdown had a massively negative effect on the mental health of the population in Nepal. The increased rates of suicide and self-harm also simultaneously exposed the great pre-existing fragility of the mental health of the nation. It is therefore vital that both Nepal and other countries alike take cognizance of the fact that extra support and preventative measures need to be introduced during this difficult period and that further national programs must be employed to best aid the mental health of their fellow countrymen.
